# Detectability of Absorption and Reduced Scattering Coefficients in Frequency-Domain Measurements Using a Realistic Head Phantom

**DOI:** 10.3390/s130100152

**Published:** 2012-12-24

**Authors:** Xiaofeng Zhang, Andrew Webb

**Affiliations:** 1 Department of Radiology, Duke University Medical Center, DUMC 3808, Durham, NC 27710, USA; 2 Department of Radiology, Leiden University Medical Center, Albinusdreef 2, 2333 ZA Leiden, The Netherlands; E-Mail: a.webb@lumc.nl

**Keywords:** near infrared, absorption, scattering, fast signal, detectability, sensitivity, frequency domain, phantom, human head

## Abstract

Detection limits of the changes in absorption and reduced scattering coefficients were investigated using a frequency-domain near-infrared system in a realistic head phantom. The results were quantified in terms of the maximum detectable depth for different activation volumes in the range of 0.8–20 microliters. The non-linear relation between the maximum detectable depth and the magnitude of changes in the absorption coefficient conform well with the Born approximation to the diffusion equation. The minimal detectable changes in the reduced scattering coefficient measured in terms of the phase signal were found to be approximately twice as large as that of the absorption coefficient using the AC signal for the same volume and at the same depth. The phase delay, which can be used to quantify the fast neuronal optical response in the human brain, showed a linear dependence on the reciprocal of the reduced scattering coefficient, as predicted by the Rytov approximation.

## 1. Introduction

Near infrared (NIR) spectroscopy and imaging methods are increasingly important measurement tools in biology, neuroscience, and medicine because of their noninvasive nature, high chemical specificity, high temporal resolution, high versatility, and portability [1–10]. These methods have shown great potential for human and animal functional studies when integrated with other imaging modalities such as magnetic resonance imaging, which provides structural information and enables improved signal localization and accurate image registration [11–17].

In human functional studies, NIR techniques are most often applied to measure the hemodynamic response in the brain, which peaks approximately four to six seconds after the actual neuronal response via measurement of the change in tissue absorption coefficient (*μ_a_*). It has also been shown that “fast” optical signals [18,19] can be measured using frequency-domain techniques. This fast signal is derived from the change in signal modulation phase and is believed to be associated with the reduced scattering coefficient (*μ*′*_s_*). A number of groups have reported fast signals in noninvasive human studies in terms of the changes in either the phase or the intensity of the optical signal [20–23]. Although invasive human and animal studies have demonstrated close coupling of the fast optical signal and neuronal response [19,24–26], detectability of the fast signal in non-invasive human studies is challenging [27]. This is primarily because the signal-to-noise ratio (SNR) of the fast signal is much lower than that of the conventional hemodynamics-induced signal.

To address the issue of signal detectability, we investigated the relative detection limits of the AC and DC components in frequency-domain measurements with respect to Δ*μ_a_* and the phase change corresponding to Δ*μ*′*_s_* in an imaging phantom. Similar to the concept of “contrast-detail analysis” for optical imaging described in [28], we characterized the detection limits using three parameters: the amplitude, the size, and the depth of the simulated activation. To the best of our knowledge, this type of data is not available in the literature, which is highly important to understand the fundamental characteristics of frequency-domain NIR methods (including spectroscopy and tomography) in human cerebral functional studies.

## 2. Experimental Section

Several groups have reported imaging phantoms for NIR studies, e.g., [29–34]: in most of those phantoms, different types of absorbers (e.g., ultra-fine carbon powder, ink, or dye) and scattering materials (e.g., titanium dioxide, polystyrene micro-spheres, fat emulsion, or milk) were typically dispersed in rigid, deformable, or liquid media (e.g., silicone, paraffin, polyvinyl alcohol, or water). As shown in the studies by Gibson *et al.* [29,35], realistic head phantoms are less susceptible to modeling errors arising from the cerebrospinal fluid (CSF) layer than simple semi-infinite or spherical homogenous phantoms.

**Figure 1.**
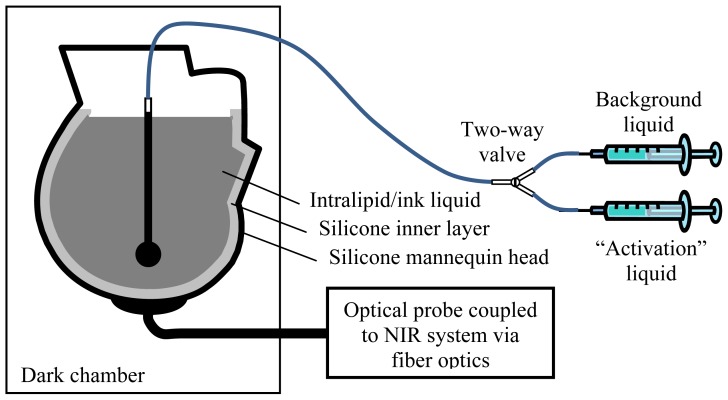
Schematic of the head phantom and experimental setup.

We constructed a realistic human head phantom from a life-sized mannequin head (Figure 1). The outer layer of the phantom was covered in cosmetically tinted silicone with a thickness of 3 mm, which simulated the scalp. Its optical properties were measured to be *μ_a_* = 0.015 and *μ*′*_s_* = 1.3 mm^−1^ at 690 nm, determined iteratively using a two-layered model as described in [36]. A layer of silicone (semi-translucent due to micro-bubbles in the material) was coated on the inner surface of the head phantom to simulate the skull and the CSF collectively. This layer of silicone had an intentionally created rough inner surface (with irregular ripples and ridges) with a thickness ranging from 2 to 6 mm.

The brain tissue was simulated using a liquid phantom consisting of 1% Intralipid (VWR, West Chester, PA, USA), 60 ppm India ink, and water. The optical properties of the liquid phantom and the semi-translucent silicone layer were measured using the method detailed in [37]: *μ_a_* = 0.011 and *μ*′*_s_* = 1.4 mm^−1^ for the liquid; and *μ_a_* = 0.0017 and *μ*′*_s_* = 0.11 mm^−1^ for the silicone layer at 690 nm. The optical probe was positioned on the head phantom at the location corresponding to the primary sensorimotor cortex. During the experiments, the head phantom was kept in a dark chamber.

**Figure 2.**
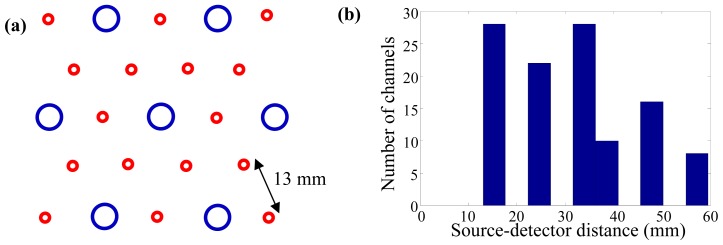
The optical probe consisted of sixteen source and seven detector fibers that were fixed in a slightly curved rubber base: (**a**) the triangular topology of the fibers: the larger/blue circles represent the detectors and the smaller/red circles the sources; and (**b**) a histogram of the source-detector distance distribution.

The optical probe was constructed by fixing the source and the detector fibers to a curved black rubber base. The rubber base was attached a hard plastic frame, thereby maintaining its shape and sour-detector separations. The topology of the fibers was designed to represent a typical functional diffuse optical tomography (DOT) experiment in which an even coverage of the region of interest (ROI) would be desired. Sixteen source fibers (each coupled to a 690-nm laser diode) and seven detector fibers bundles (each coupled to a photomultiplier tube) were arranged in a triangular pattern, Figure 2(a). The source-detector distances of the optical probe ranged from 13 to 60 mm, Figure 2(b).

We used a frequency-domain NIR spectroscopy/imaging system (Imagent, ISS, Champaign, IL, USA). The light sources were laser diodes (690 nm), which were time-multiplexed, amplitude-modulated (150 MHz), and coupled to multimode fiber optics (core size 400 μm, Thorlabs, Newton, NJ, USA). The detection fiber optic bundles (diameter 3 mm, numerical aperture 0.55, Sunoptic Technologies, Jacksonville, FL, USA) were coupled to the photomultiplier tubes (PMT), where the optical signal was converted into an electrical signal. The electrical signal was then demodulated using a heterodyne method with a cross-correlation frequency of 10 kHz, and subsequently digitized by a 16-bit analog-to-digital converter (ADC) (PCI-416M2, DATEL, Mansfield, MA, USA). The effective sampling rate for each measurement channel was 10 Hz.

We simulated functional activation using thin-walled quartz spheres (Wilmad Lab Glass, Buena, NJ, USA). Baseline condition was simulated by filling the spheres with the background liquid phantom, whereas activation condition was simulated by filling with the same type of liquid phantom but with altered optical properties by adjusting the amount of ink and Intralipid. The temporal feature of the functional activation was simulated by switching between the background and altered liquids. Five spheres of different volumes (0.8, 2.1, 5.2, 11, and 20 mL, *i.e.*, inner diameters 12, 16, 21, 28, and 34 mm) were placed at different distances, which were measured from the bottom of the sphere to the outer surface of the head phantom, in 2-mm increments. One sphere was placed in the head phantom in any given measurement. Two syringes were connected to a two-way valve and used to fill and drain the sphere: one with background liquid for baseline and another with altered optical properties for activation (Figure 1). Human cerebral functional activation was simulated by switching the liquids every 10 s for a total of 20 times, *i.e.*, 10 activations and 11 resting intervals interleaved in a total experimental time of 210 s. Similar experimental paradigms are typically used in human studies because it was an appropriate compromise between the amount of acquired data and minimizing subject motion.

For each source-detector measurement channel, the data were first folding-averaged and then processed using the following criteria to determine the detectability: (a) the absolute value of the correlation coefficient of the averaged signal (300 time-series sampling points) and the boxcar activation pattern should be greater than 0.3 (equivalent to a significance value of *p* < 2 × 10^−7^, according to [38]); and (b) the SNR of the processed signal should be >0.5. The SNR was defined as the ratio of the relative change in signal intensity to the standard deviation of the baseline signal. In this study, the detectability was investigated based on the raw data, *i.e.*, the AC, DC, and phase signals. Activation was considered detectable for a given configuration if at least one measurement channel met both of the above criteria.

We used five different liquids that produced 5, 10, 15, 20, and 30% increase of *μ_a_* with respect to the background. This range of Δ*μ_a_* values was chosen to cover the normal physiological conditions derived from published experimental data [12,17,39–47] and values adopted by other phantom and simulation experiments pertaining to human functional studies [48–52]. We did not find any experimental data regarding the values of Δ*μ*′*_s_* for functional brain studies in the literature. In a simulation study, the authors estimated the value of Δ*μ*′*_s_* being less than 0.4% using a “proportionality factor” assuming a semi-infinite or layered medium [27]. Reported experimental measurements of the phase delay in fast signal measurement range from 0.7 to 10 ps, as summarized by Steinbrink *et al.* [27], and would correspond to much larger values of Δ*μ*′*_s_*. It should be noted that values of the phase delay are very sensitive to the size and the depth of the activation, as well as the sensitivity profile of the optical probe, which should be taken into consideration when comparing experimental results from different subjects and in different cortical areas. In our phantom study we used values of Δ*μ*′*_s_* (10, 20, 30, 40, and 50% increase from baseline) that produced detectable changes in the phase of the optical signal for the same size and depth of activation, sampling rate, and the number of measurements used to measure the detectability of Δ*μ_a_*.

## 3. Results and Discussion

**Figure 3.**
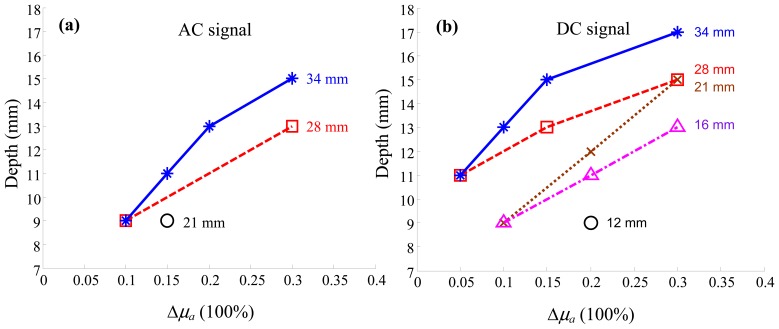
Detectability of the changes in absorption coefficient *μ_a_* in terms of (**a**) the AC signal and (**b**) the DC signal, with respect to the background (baseline) value.

The maximum detectable depth is plotted against the relative value of Δ*μ_a_* (percentage change) for different activation volumes in terms of the AC and the DC signals, Figure 3(a,b), respectively. It was obtained by incrementally increasing the depth of activation, measured from the bottom of the inner surface of the sphere to the outer surface of the phantom, until the change in *μ_a_* or *μ*′*_s_* was undetectable according to the criteria described previously. As expected, the detectable depth of activation increases as the size and the amplitude of change increase. It is noteworthy that because the sensitivity function is narrower for superficial measurement channels than deeper channels, the depth-Δ*μ_a_* curves appear non-linear. This effect is more apparent for larger spheres than smaller ones.

Following the theoretical analysis in [53,54], the first-order Born solution of the diffusion equation is given by:

(1) $$\delta U=-\int_{\Omega }\delta \mu_{a}G_{0}U_{0}d\Omega$$

where *U*_0_ is the fluence of the light source (*i.e.*, the field of light radiation) in the baseline condition, *G*_0_ is the Green function, *U* is the detected photon fluence rate, and *Ω* is the integral volume. It shows that the change in the optical signal is linearly related to Δ*μ_a_* if scattering can be ignored: this corresponds to measurement of the hemodynamic optical signal, as described previously.

The SNR of the optical signal is linearly related to the amplitude, but its relationship to the volume and the depth of Δ*μ_a_* is non-linear since the integral kernel *G*_0_*U*_0_ is spatially inhomogeneous. This is evident in the data shown in Figure 3, in which the dependence of the maximum detectable depth (y-axis) on the minimum detectable Δ*μ_a_* (x-axis) is non-linear for a given activation volume. However, such non-linearity stems from the non-uniform distribution of the sensitivity function. For smaller spheres at smaller depth, the non-uniformity is much less than that of larger spheres at larger depth. As a result, the depth-detectability relations for smaller spheres (also smaller detectable depth) appear linear under our experimental conditions.

Comparing Figure 3(a,b), the result indicates that activations are more sensitive using the DC signal than using the AC signal: smaller volumes of activation being detectable using the DC signal; and the maximum detectable depth using the DC signal being approximately 2–3 mm larger than using the AC signal for a given volume.

Another observation from Figure 3 is that, although the detectable depth using the DC signal is only slightly larger than that using the AC signal, the DC signal is capable of detecting much smaller activations in our experimental setup: activation volumes of 0.8 and 2.1 mL were not detectable using the AC signal even at the smallest depth (10 and 12 mm respectively from the bottom of the inner surface of the activation to the probe/phantom interface).

**Figure 4.**
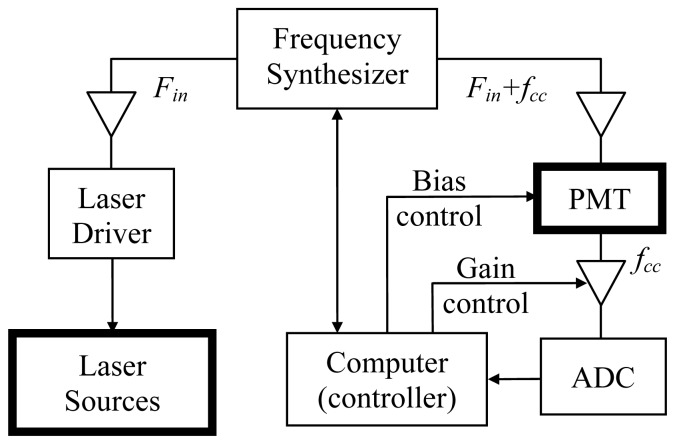
Schematic of the frequency-domain NIR system, where *F_in_* is the modulation frequency and *f_cc_* is the cross-correlation frequency.

Although absolute values of detectability of the AC and DC signals depend upon the particular measurement system, the frequency dependence, for example, of relative detectability can be estimated by considering the noise contributions to each measurement. A schematic of our NIR system is shown in Figure 4. In phantom experiments, the only source of noise is “system noise” which has several contributors: quantum noise from the photodetector when the intensity of light is low, dark noise due to the dark current in the photodetector (PMT), thermal noise from electronics (particularly the signal amplifiers), quantization noise from the ADC, temperature drift (causing the changes in the semiconductor characteristics and the level of thermal noise) as well as the fluctuation of the voltage/current supplies of the PMT and the laser diodes. The noise level at the input to the ADC is dictated by the characteristics of each electronic component. If one assumes that the noise figure and the gain of the amplifiers are frequency-independent across the measurement bandwidth (10 kHz in this study), then the system noise level seen by the ADC is the same for both the AC and the DC measurements since the two measures are not separated until after quantization at the ADC. In our system, the noise in the AC and the DC signals is dominated by the analogue data acquisition subsystem, because the amplifier gain is high, the noise figure is relatively low, and the high digital resolution of the 16-bit ADC (a lower limit of the quantization error of <1 × 10^−5^).

**Figure 5.**
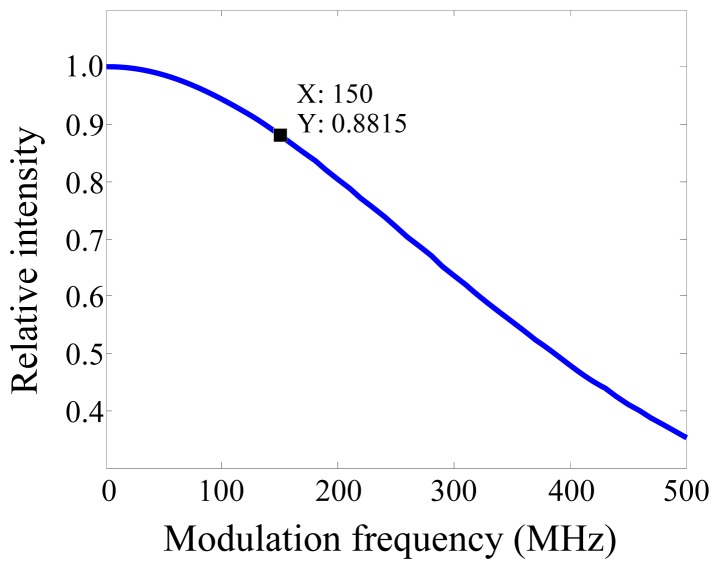
Frequency-dependence of relative sensitivity for a semi-infinite medium (*μ_a_* = 0.01 and *μ*′*_s_* = 1 mm^−1^) with a source-detector distance of 20 mm at a depth of 20 mm.

The relative SNR of the AC and the DC signals are therefore determined both by their relative intensities and relative sensitivities. In terms of relative intensities, the modulation depth (defined as the ratio of AC to DC signals) of the NIR system used in our experiment was 50%, meaning that the DC signal is twice as high as the AC at the source. The relative sensitivity depends on the modulation frequency of the instrumentation. The frequency-dependence of the sensitivity function for a semi-infinite medium (using the analytical expression in [55]) is shown in Figure 5. For a source-detector separation of 20 mm, at a modulation frequency of 150 MHz, and at a depth of 20 mm from the surface mid-way between the source and the detector, the absolute value of the sensitivity function is 88% of its value at DC. The above analysis indicates that at 150 MHz modulation, the DC signal should be (50% × 88%)^−1^ = 2.3 times more sensitive than the AC signal in terms of SNR. Note that at higher modulation frequencies, this advantage will increase. In practice, one also has to consider that at higher frequencies electronic components typically have lower gain and higher noise figure.

**Figure 6.**
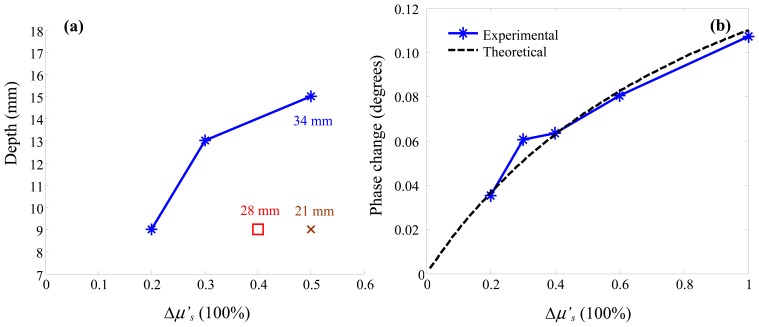
(**a**) Detectability of the change in the reduced scattering coefficient from the phase signal and (**b**) relationship between the changes in the phase signal and the values of the reduced scattering coefficient revealed in experimental data and theoretical analysis.

The detectability of Δ*μ*′*_s_* in terms of changes in the phase signal is shown in Figure 6(a). Both Δ*μ_a_* and Δ*μ*′*_s_* result in changes in the AC and the DC signals, but the phase signal is solely dependent on Δ*μ*′*_s_*: a significant advantage of the frequency-domain measurement method [56]. The detectability of Δ*μ*′*_s_* shows similar characteristics to that of Δ*μ_a_*. However, comparison between Figures 3 and 6(a) shows that, to achieve a similar maximal detectable depth, the amplitude of Δ*μ*′*_s_* has to be larger than that of Δ*μ_a_* by a factor of ∼2.

In order to understand the shape of the graph in Figure 6, we note that the first-order Rytov solution to the diffusion equation:

(2) $$\delta \phi =\frac{1}{U_{0}}\int_{\Omega }\delta D\left(\nabla G_{0}\cdot \nabla U_{0}\right)d\Omega$$

in which *ϕ* is defined as *ln*(*U*). It shows that *δϕ* is linearly related to the change in the light diffusion coefficient *D*, if the changes in the absorption coefficient are ignored (a reasonable assumption since immediately after neuronal activation the hemodynamic response has not yet developed). As the fluence is complex and can be written in the form of *U* = *A* · *e^jθ^*, it follows that:

(3) $$\delta \phi =ln\left(U\right)-ln\left(U_{0}\right)=ln(A\cdot e^{j\theta })-ln\left(A_{0}\cdot e^{j\theta_{0}}\right)=ln\;\left(\frac{A}{A_{0}}\right)+j\left(\theta -\theta_{0}\right)$$

Since the diffusion coefficient can be defined as *D* = (3*μ*′*_s_*)^−1^, the dependence of the phase delay of the optical signal should be a linear function of the reciprocal of *μ*′*_s_* in the form of *y* = *a*(1 − *x*^−1^), where *y* is the phase delay, *x* is the normalized value of *μ*′*_s_* (by its baseline value, 1 < *x* < 2), and *a* is a constant. Figure 6(b) shows good agreement between the experimentally measured phase delay (34 mm sphere at depth 11 mm) and the theoretical prediction (the fitted value of 0.22 in this instance).

**Figure 7.**
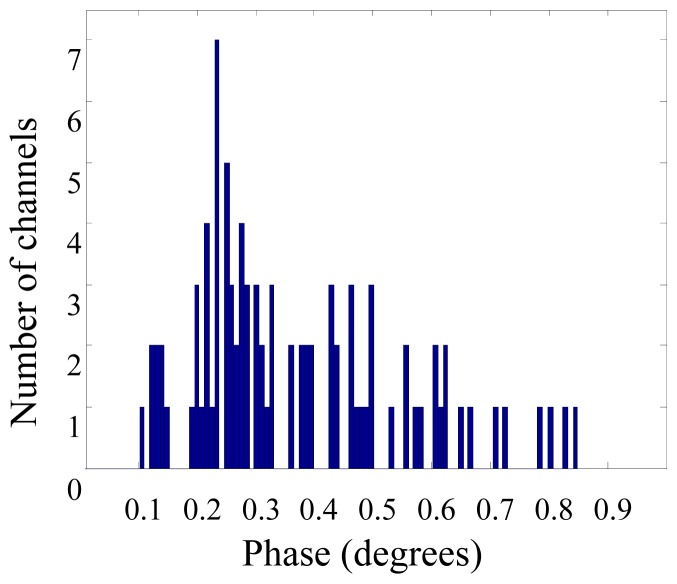
A histogram of the standard deviation of the phase signal from a representative measurement. The channels that had AC signals less than 5% of the maximum value were excluded to eliminate unreliable phase measurements due to small signal intensity.

Detectability of Δ*μ*′*_s_* is fundamentally limited by the SNR of the phase signal. The dominant noise source for phase measurement is the time-jitter of the ADC, which is caused by instabilities in the core frequency source (master oscillator). In our system, the ADC used an external frequency synthesizer (D620, PTS, Littleton, MA, USA) as the frequency source (10 MHz ± 2 Hz). For a cross-correlation frequency of 10 kHz, this tolerance is equivalent to a peak-to-peak phase jitter of ±0.72°, which corresponds to a standard deviation of 0.42° if the jitter is truly random. Figure 7 shows a histogram of the standard deviation of the phase signal from a representative measurement. About 65% of the measurement channels had standard deviations equal or less than the error limit estimated above. Even the largest value is only twice the estimated error limit.

To ensure the validity of our general conclusions, we intentionally chose a set of relatively conservative criteria to define the detectability. As a result, some measurements were determined undetectable by these particular criteria, but were in fact detectable by visual inspection. In addition, the number of valid measurement channels had impact on the detectability. Our experiment had 112 measurement channels (16 sources and 7 detectors). The number of valid channels for superficial activations (*i.e.*, short distance) is significantly larger than deep activations (longer distance). As a result, these limitations influenced the *observed* detectability by means of introducing false negative in the results (omitting detectable activation). However, the data sets that passed the relative conservative detectability criteria are robust and well support the general conclusions based upon them.

## 4. Conclusions

We have presented results using a life-size head phantom with realistic optical properties for studying the measurement sensitivity of absorption and reduced scattering coefficients. The detectability of simulated functional activation was quantified in terms of the maximum detectable depth for absorption and scattering changes of different amplitudes and volumes ranging 0.8–20 microliters using a frequency-domain NIR system with a modulation frequency of 150 MHz. The non-linear relation between the maximum detectable depth and the magnitude of changes in the absorption coefficient conform well with the Born approximation to the diffusion equation. The amplitude of the experimentally measured phase delay was found to be linearly related to the reciprocal of *μ*′*_s_* as predicted by the first-order Rytov solution to the diffusion equation. For the same volume and depth of activation, the minimum detectable amplitude of Δ*μ*′*_s_* is approximately twice as large as that of Δ*μ_a_*.
